# The case for a regional approach to publication impact

**DOI:** 10.3332/ecancer.2018.ed78

**Published:** 2018-01-29

**Authors:** Stevan Bruijns, Camillo Lamanna

**Affiliations:** Faculty of Health Sciences University of Cape Town, Anzio Road, Cape Town, 7935, South Africa

**Keywords:** scholarly publishing, low-income population, impact metric

## Abstract

Healthcare-related research is largely regional. Put simply, this is because disease burdens differ between world regions. Even global burdens, such as ischaemic heart disease and cancer, display distinctive characteristics in certain regions that are not seen in others. Regional differences in infrastructure, resources and human capital further compound the differences seen, as they affect the way in which the local scientific community can interact with the local disease burden. As such, it seems fair to assume that healthcare-related research ought to be regionally distributed. Although translation of research between regions can sometimes be done, the larger the gap in infrastructure, resources or human capital between regions, the less likely it is that it can be adequately bridged. A recent example of this pertains to accepted life-saving treatment for sepsis in high-income settings, which had the opposite effect when implemented and evaluated in low-income Zambia. This regionality of clinical medicine is, however, not reflected in academic publishing; the impact of a journal is measured and understood by metrics that use the world as their denominator. Therefore, top medical journals are perceived to be relevant equally to all contexts and regions. However, there is a strong case to be made that this lack of granularity is deleterious, and that the creation of a regional impact metric would place clinicians, researchers, and libraries in a better position to understand which journals are relevant to their context and practice.

Healthcare-related research as we know it today started out during the mid-seventeenth century with a distinct focus on the individual. [1, 2] The preferred publication format at the time was the case report (ironically the least preferred today). [3] However, an increased understanding of pathophysiology followed during the mid-nineteenth to early twentieth centuries. This led to the growing recognition that differing underlying disease processes were responsible for the various pathologies identified in case reports. The result was the establishment of various specialist journals, with a focus shift from the individual towards pathology. [3] Then, during the twentieth century, development of newer medicines to treat pathologies—combined with novel evaluation techniques (such as the randomised controlled trial)—shifted healthcare-related research from pathology towards therapy. In those early days, the truths of medicine were largely considered universal, a belief that likely originated during the Enlightenment (eighteenth century) which proclaimed that all men were equal. [4] In other words, if given the same therapy, for the same disease, the response surely should be equal too. Healthcare journals were considered on a par with the natural science journals alongside which they were born. [2] Although the latter did convey universal, region-independent facts about the natural world, the former were unable to do so. The philosophical belief in universal equality simply did not extend to equality of response to treatment. During the second half of the twentieth century, evidence suggested time and again that what worked in one context did not always work so well in another (the Zambia sepsis study is a more recent example). [5] Given growing disparities in health resources, what works in a high-income context is not necessarily feasible in lower income contexts—or the majority of the global population.

While there are good reasons to consider healthcare-related research in context, the current architecture of healthcare publishing is such that findings are presented as global. Arguably, this would be less of an issue if the research outputs of low- and middle-income countries were on a par with those of high-income countries. If Tanzanian-based physicians, say, could practice medicine using an evidence base of similar size and quality to that of, say, an England-based physician, it is unclear what the extra benefit of describing regional research impact would be. However, this is not the situation. There are various reasons why research from low- and middle-income countries fails to assert itself on a global scale. Poor English language skills, lack of experience in applying the research method, lack of funding, are all surely part of the problem. [6] Each of these is a challenge in its own right, none of which is likely to be resolved overnight. However, there are also challenges that can be readily addressed: currently, researchers, academic libraries, and authors from low- and middle-income countries struggle to discern which journals have impact in their respective audiences—a regional impact metric would go some way to rectifying this.

Impact is a challenging theme. Increasingly, researchers have begun to question the hegemony of the Impact Factor, with a multitude of rival metrics suggested in recent years. [7, 8, 9] Moreover, it seems that its basic measure of impact—the citation—does not align well with what researchers in clinical medicine or practising clinicians would consider impact; that is, changing real-world clinical practice. For example, of the ten top-cited articles ever published by *The New England Journal of Medicine*, three are trials of monoclonal antibody anti-cancer drugs that are largely unaffordable in low- or middle-income countries. [10] Seeing that low- and middle-income countries make up around 82% of the global population, these articles are unlikely to have the global impact suggested by their citations. Therefore, although citations would no doubt contribute to a regional impact metric, it seems unlikely that they would end up as its foundation in the same way as they are the foundation of the Impact Factor.

Another measure, which intuitively appears more reflective of research dissemination, is views. It is a little-known fact that at the individual article level, there is almost no correlation between publication citations and views. [11] Odd as this may seem at first, it does make sense. Scholarly impact (citations) refers to the use of research to generate new research, whereas disseminative impact (views) refers to the use of research to improve knowledge. Some research leans towards a scholarly impact rather than a disseminative impact (and vice versa), but seldom both. As scholarly impact metrics like Impact Factor (citations-per-publication) are far better-known than disseminative impact metrics like views-per-publication, journals with high scholarly impact tend to dominate global rankings. Furthermore, there are tangible barriers to the dissemination of research that are not captured directly by any existing metric. Cost and access are a few notable examples. Cost is an important variable as many low- and middle-income researchers are unable to afford the access charges to subscription articles. Compared to an American researcher, the out-of-pocket expense to access a subscription article is 1.6 times higher for a Brazilian researcher, 2.3 times higher for a Turkish researcher and 3.5 times higher for a Ghanaian researcher. [12, 13] The same ratios apply to the processing charges that allow paid open access publication of regionally important research (also referred to as gold- or hybrid- open access). Although access through self-archiving of articles (also referred to as green open access) provides a low cost access alternative, journals vary widely in their policies on how this can be applied. It seems reasonable to posit that the absence of a disseminative journal ranking has given journals no incentive to increase global access; therefore, one step to addressing access may be to include such a measure within a regional impact metric.

At this point, a journal editor or publisher could say this is all too quick: the function of a journal is to employ a robust mechanism of validation via editorial and peer review to ensure a certain standard of scholarship in what it publishes. This argument is valid but also contains within it inherent hidden biases. We may liken the publication process to a job interview. *Prima facie*, if an interviewer asks all applicants the same questions and grades everyone on the same scoresheet, then they cannot be accused of bias. But what if some applicants don’t have the training to sell themselves on their CV so never get to interview? What if some applicants cannot afford the fare to get to the hiring office? What if someone from a different background does not use the vernacular expected in interview situations and scores more poorly as a result? These are all hidden biases that are also present in journal publishing which could be mitigated by: offering additional research method and statistical support to researchers from low- and middle-income countries, offering reduced open-access fees to authors from these settings, and offering language assistance to those who do not have English as their first language. Some journals already offer some of these, however it is by no means the standard.

Devising new metrics that respect the regionality of healthcare, and acknowledge the difference between scholarly and disseminative impact, is an important step towards equity in healthcare-related research. Using existing available data to produce regional impact rankings should technically be easy. This may in turn incentivise top (largely Western) journals to adopt some of the aforementioned measures to increase their regional impact in low- and middle-income countries. Differentiating between disseminative and scholarly impact may incentivise journals further to widen access to these countries. Moreover, such metrics can help researchers identify the journals that will provide the highest uptake of their (regional) work, can provide clinicians with the most relevant research in their regions, and enable librarians to save costs by subscribing to a core list of journals that have proven impact within their region. Only a regional view of impact can reveal these nuances and unlock the regional benefits they may present. Newly-launched initiatives such as the Journal Publishing Practices and Standards for journals from the global South has already taken a stab at defining quality on a regional scale. [14] More work is needed to derive, refine and promote reliable regional impact metrics that can help drive quality within non-Western regions.
